# Crystal structure of (1*S*,2*R*)-2-hy­droxy-1,2-di­phenyl­ethan-1-aminium (*S*)-2-aza­niumyl­butane­dioate monohydrate

**DOI:** 10.1107/S2056989017015729

**Published:** 2017-11-03

**Authors:** Isao Fujii

**Affiliations:** a School of Science, Tokai University, 4-1-1 Kitakaname, Hiratuka, Kanagawa 259-1292, Japan

**Keywords:** crystal structure, diastereomeric salt separation, zwitterion, inter­molecular hydrogen bonding, helical columnar structure

## Abstract

The title diastereomeric salt, formed between 2-amino-1,2-di­phenyl­ethanol (ADE) and aspartic acid (ASP), C_14_H_16_NO^+^·C_4_H_6_NO_4_^−^·H_2_O, crystallizes as a monohydrate. The 1,2-di­phenyl­ethyl group in the cation has a *cis* conformation, and the aspartic acid anion is in the zwitterionic form. In the crystal, the ASP anions are linked *via* N—H⋯O hydrogen bonds to form a 2_1_ helix along the *b*-axis direction. The helices are linked by the ADE cations *via* O—H⋯O and N—H⋯O hydrogen bonds, forming layers parallel to the *bc* plane. There are channels in the layers that are occupied by water mol­ecules, which link to both the anions and cations *via* O_water_—H⋯O and N—H⋯O_water_ hydrogen bonds. There are also C—H⋯O and C—H⋯π inter­actions present within the layers.

## Abstract 2

The title diastereomeric salt, formed between 2-amino-1,2-di­phenyl­ethanol (ADE) and aspartic acid (ASP), crystallizes as a monohydrate. In the crystal, the ASP anions are linked *via* N—H⋯O hydrogen bonds to form a 2_1_ helix along the *b*-axis direction.

## Chemical context   

The production of chiral compounds has great importance in the pharmaceutical industry, and diastereomeric salt separation is still widely applied in the process. A synthetic optical resolving agent, chiral 2-amino-1,2-di­phenyl­ethanol (ADE) (Read & Steele, 1927 ▸), has been widely tried and used in diastereomeric salt-separation methods for chiral alcohols or organic acids. l-(*S*)-aspartic acid (ASP) is a known neurotransmitter, and d-(*R*)-ASP is a non-essential amino acid, one of two d-amino acids commonly found in mammals. d-ASP has also attracted attention as residue in the anti­fungal bacitracin, while *N*-methyl-d-aspartic acid (NMDA) acts as a specific agonist at the NMDA receptor. d-amino acids are mainly resolved enzymatically with d-amino­acyl­ase (EC 3.5.1.14) in industrial applications. The optical separation of ASP with *cis*-ADE was introduced without chemical modification. The crystal structure of the title mol­ecular salt, formed between l-(*S*)-ASP and (*1R,2S*)-*cis*-ADE, is reported herein.
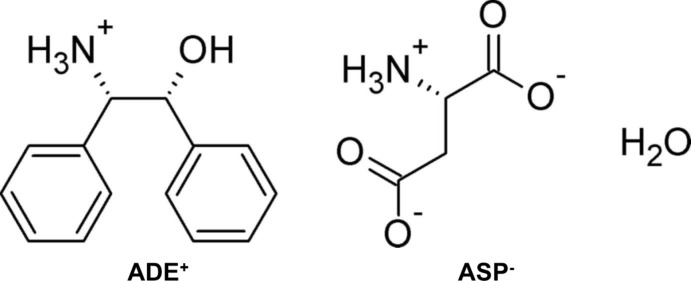


## Structural commentary   

**Figure 1:**
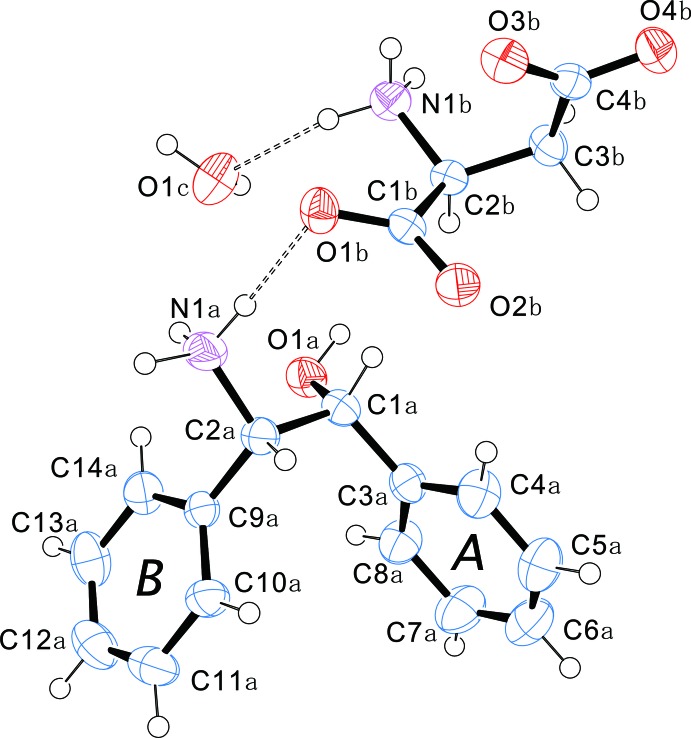
A view of the mol­ecular structure of (1*S*,2*R*)-*cis-*ADE·(*S*)-ASP monohydrate, with the atom and ring labelling. Displacement ellipsoids are drawn at the 50% probability level. Dashed lines indicate the hydrogen bonds (see Table 2 ▸).

The mol­ecular structures of the components of the title salt are shown in Fig. 1 ▸, and selected torsion angles are given in Table 1 ▸. It can be seen that the hy­droxy and protonated amino groups of *cis*-ADE form a tweezer-like motif. The dihedral angle between the phenyl rings (*A* and *B*; Fig.1) is 48.71 (9)° and the torsion angle O1*A*—C1*A*—C2*A*—N1*A* is −65.0 (2)°. The hy­droxy group adopts a *gauche* conformation [O1*A*—C1*A*—C2*A*—C9*A* = 60.1 (2)°] with respect to phenyl ring *B*. Thus, the tweezer-like motif is twisted with respect to the phenyl groups. This arrangement is similar to that found in racemic *cis*-ADE (Fujii, 2015 ▸) and the diastereomeric salts formed with *cis*-enanti­omers.

l-(*S*)-ASP crystallizes as a deprotonated zwitterion. The succinate group adopts a *cis* conformation [C1*B*—C2*B*—C3*B*—C4*B* = −53.0 (2)°], which is the motif commonly found in l-ASP salts; for example l-His·l-ASP monohydrate (Suresh & Vijayan, 1987 ▸). The amino and residual carb­oxy groups have a slightly right-handed helical-shape; torsion angles N1*B*—C2*B*—C3*B*—C4*B* and C2*B*—C3*B*—C4*B*—O3*B* are 73.0 (2) and 1.4 (3)°, respectively.

## Supra­molecular features   

**Figure 2:**
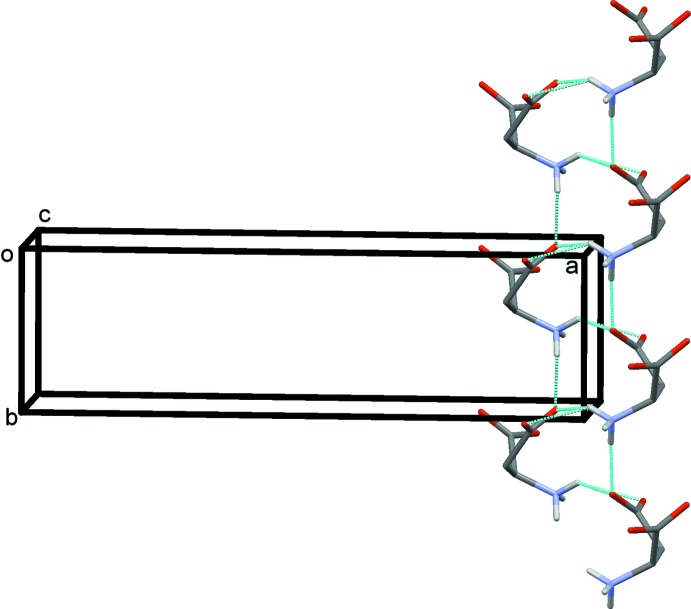
A view along the *c* axis of the right-handed *2_1_*-helix of ASP anions. Hydrogen bonds are shown as dashed lines (see Table 2 ▸) and C-bound H atoms have been omitted.

**Figure 3:**
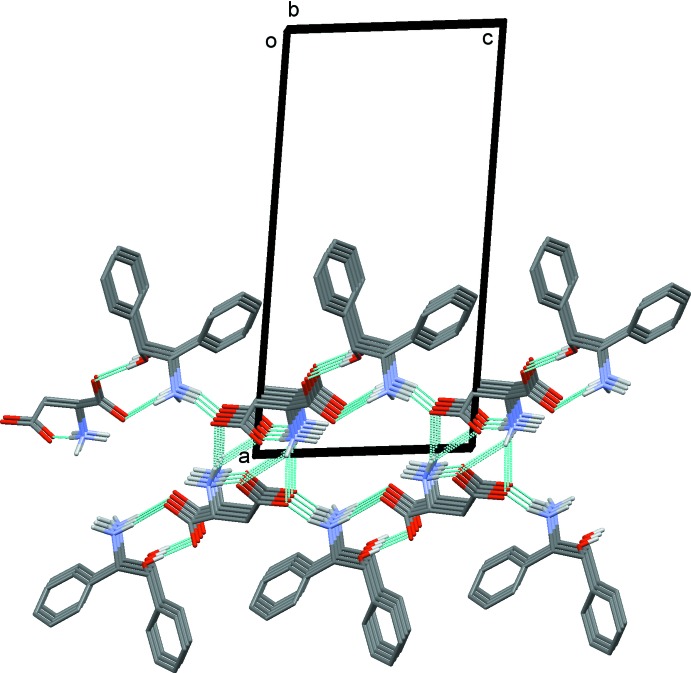
A partial view along the *b* axis of the crystal packing of the ASP helices linked by the ADE cations. Hydrogen bonds are shown as dashed lines (see Table 2 ▸) and C-bound H atoms have been omitted.

**Figure 4:**
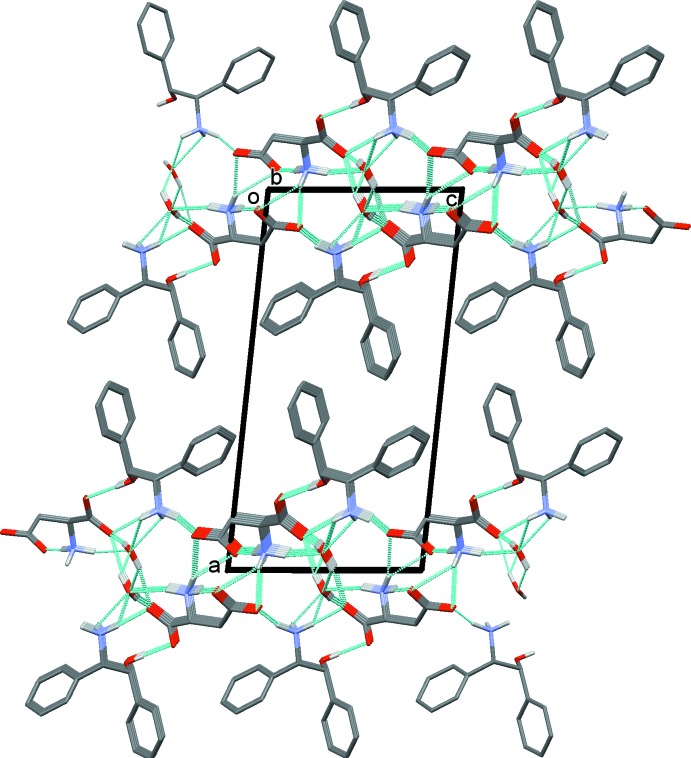
A view along the *b* axis of the crystal packing of (1*S*,2*R*)-*cis-*ADE·(*S*)-ASP monohydrate. Hydrogen bonds are shown as dashed lines (see Table 2 ▸) and C-bound H atoms have been omitted.

In the crystal, the (*S*)-ASP anions correlated with crystallographic symmetry are linked *via* N1*B*—H1*B*3⋯O4*B* ^ii^ [2.868 (2) Å] hydrogen bonds into *C*(6) chains to form a right-handed *2_1_*-helix along the *b*-axis direction (Fig. 2 ▸ and Table 2 ▸). The helices are linked by the (*1R,2S*)-*cis*-ADE cations *via* N—H⋯O hydrogen bonds [N1*A*—H1*A*2⋯O1*B* = 2.862 (2) Å and N1*A*—H1*A*1⋯O4*B* ^iii^ = 2.742 (3) Å] and O—H⋯O hydrogen bonds [O1*A*—H1*O*1⋯O2*B* ^i^ = 2.752 (2) Å], forming layers parallel to the *bc* plane (Fig. 3 ▸, Table 2 ▸). There are channels in the layers that are occupied by water mol­ecules which link to both the anions and cations *via* tetra­hedrally placed hydrogen bonds; O_water_—H⋯O hydrogen bonds [O1*C*—H1*OB*⋯O1*B* ^i^ = 2.734 (2) Å and O1*C—*H1*OA*⋯O1*B* ^iv^ = 2.840 (2) Å] and N—H⋯O_water_ hydrogen bonds [N1*B*—H1*B*2⋯O1*C* = 2.938 (2) Å and N1*A*—H1*A*3⋯O1*C* = 2.926 (3) Å], shown in Fig. 4 ▸; see also Table 2 ▸. There are also C—H⋯O and C—H⋯π inter­actions present within the layers (Table 2 ▸). Finally, the hydro­phobic and hydro­philic layers are well separated along the *a*-axis direction.

## Database survey   

The author has reported the crystal structures of several amino acids without chemical modification including the chiral resolving agents; 1,1′-bi­naphthalene-2,2′-diyl hydrogen phosphate, 2-phen­oxy­propionic acid and mandelic acid (Fujii & Hirayama, 2002 ▸; Fujii *et al.*, 2005 ▸, 2006 ▸). The crystal structures of racemic *trans*- and *cis*-ADE have been reported (GAQXON: Bari *et al.*, 2012 ▸; RUTROP: Fujii, 2015 ▸, respect­ively). Recently, the solvent-induced chirality switching in optical resolution between mandelic acid and *cis*-ADE has been demonstrated (Shitara *et al.*, 2013 ▸). Moreover, a database search (CSD Version 5.28, last update May 2017; Groom *et al.*, 2016 ▸) yielded other comparable structures, *viz.* l-aspartic acid (LASPRT: Derissen *et al.*, 1968 ▸), l-aspartic acid monohydrate (IJEQET: Umadevi *et al.*, 2003 ▸) and *N*-methyl-d-aspartic acid monohydrate (KEWGUO: Sawka-Dobrowolska *et al.*, 1990 ▸).

## Synthesis and crystallization   

(1*R*,2*S*)-*cis*-2-Amino-1,2-di­phenyl­ethanol (ADE) and aspartic acid (ASP) were purchased from Sigma–Aldrich Co. Ltd. The title mol­ecular salt was obtained from an aqueous ethanol solution of racemic-ASP and (1*R*,2*S*)-*cis*-ADE in a 2:1 molar ratio, heated to 333 K under stirring. On slow cooling to ambient temperature and slow evaporation of the solvent, colourless rod-shaped crystals were obtained.

## Refinement   

Crystal data, data collection and structure refinement details are summarized in Table 3 ▸. All the H atoms were located in difference-Fourier maps. The NH_3_^+^, OH, and water H atoms were freely refined. The C-bound H atoms were included in calculated positions and treated as riding atoms: C—H = 0.93–0.98 Å with *U*_iso_(H) = 1.2*U*_eq_(C).

## Supplementary Material

Crystal structure: contains datablock(s) global, I. DOI: 10.1107/S2056989017015729/su5398sup1.cif

Structure factors: contains datablock(s) I. DOI: 10.1107/S2056989017015729/su5398Isup2.hkl

Supporting information file. DOI: 10.1107/S2056989017015729/su5398Isup3.cml

CCDC reference: 1582706

Additional supporting information:  crystallographic information; 3D view; checkCIF report

## Figures and Tables

**Table 1:** Selected torsion angles (°)

O1*A*—C1*A*—C2*A*—N1*A*	−65.0 (2)	N1*B*—C2*B*—C3*B*—C4*B*	73.0 (2)
C3*A*—C1*A*—C2*A*—C9*A*	−66.1 (2)	C1*B*—C2*B*—C3*B*—C4*B*	−53.0 (2)
O1*B*—C1*B*—C2*B*—N1*B*	17.4 (2)	C2*B*—C3*B*—C4*B*—O3*B*	1.4 (3)

**Table 2:** Hydrogen-bond geometry (Å, °) *CgB* is the centroid of phenyl ring *B* (C9–C14).

*D*—H⋯*A*	*D*—H	H⋯*A*	*D*⋯*A*	*D*—H⋯*A*
N1*B*—H1*B*1⋯O3*B* ^i^	0.92 (4)	1.92 (4)	2.819 (2)	168 (3)
N1*B*—H1*B*3⋯O3*B* ^ii^	0.87 (3)	2.46 (3)	3.112 (2)	132 (3)
N1*B*—H1*B*3⋯O4*B* ^ii^	0.87 (3)	2.20 (3)	2.868 (2)	134 (3)
O1*A*—H1*O*1⋯O2*B* ^i^	0.88 (3)	1.88 (3)	2.752 (2)	171 (3)
N1*A*—H1*A*1⋯O4*B* ^iii^	1.08 (4)	1.70 (4)	2.742 (3)	162 (3)
N1*A*—H1*A*2⋯O1*B*	0.95 (3)	1.92 (3)	2.862 (2)	173 (3)
O1*C*—H1*OB*⋯O1*B* ^i^	0.92 (4)	1.82 (4)	2.734 (2)	173 (3)
O1*C*—H1*OA*⋯O1*B* ^iv^	0.90 (4)	2.01 (4)	2.840 (2)	153 (4)
N1*A*—H1*A*3⋯O1*C*	0.87 (4)	2.45 (3)	2.926 (3)	115 (2)
N1*B*—H1*B*2⋯O1*C*	0.96 (2)	2.01 (3)	2.938 (2)	163 (2)
C2*A*—H2*A*⋯O1*A* ^v^	0.98	2.42	3.304 (3)	150
C14*A*—H14*A*⋯O4*B* ^vi^	0.93	2.53	3.371 (3)	150
C3*B*—H3*B*2⋯CgB^iii^	0.97	2.87	3.6127 (16)	134

Symmetry codes: (i) 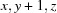; (ii) 
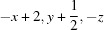
; (iii) 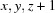; (iv) 
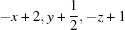
; (v) 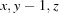; (vi) 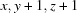.

**Table 3:** Experimental details

Crystal data	
Chemical formula	C_14_H_16_NO^+^·C_4_H_6_NO_4_^−^·H_2_O
*M*_r_	364.39
Crystal system, space group	Monoclinic, *P*2_1_
Temperature (K)	297
*a*, *b*, *c* (Å)	18.310 (8), 5.2661 (10), 9.2792 (10)
β (°)	96.070 (4)
*V* (Å^3^)	889.7 (4)
*Z*	2
Radiation type	Cu *K*α
μ (mm^−1^)	0.86
Crystal size (mm)	0.4 × 0.2 × 0.2

Data collection	
Diffractometer	Enraf–Nonius CAD-4
Absorption correction	ψ scan (North *et al.*, 1968 ▸)
*T*_min_, *T*_max_	0.74, 0.86
No. of measured, independent and observed [*I* > 2σ(*I*)] reflections	2114, 2051, 1907
*R*_int_	0.020
(sin θ/λ)_max_ (Å^−1^)	0.626

Refinement	
*R*[*F* ^2^ > 2σ(*F* ^2^)], *wR*(*F* ^2^), *S*	0.028, 0.079, 1.06
No. of reflections	2051
No. of parameters	272
No. of restraints	1
H-atom treatment	H atoms treated by a mixture of independent and constrained refinement
Δρ_max_, Δρ_min_ (e Å^−3^)	0.17, −0.13
Absolute structure	No quotients, so Flack (1983 ▸) parameter determined by classical intensity fit
Absolute structure parameter	0.1 (2)

Computer programs: CAD4 (Enraf–Nonius, 1994 ▸), *XCAD4* (Harms & Wocadlo, 1995 ▸), *SHELXS86* (Sheldrick, 2008 ▸), *SHELXL2017* (Sheldrick, 2015 ▸), *ORTEP-3 for Windows* and *WinGX* (Farrugia, 2012 ▸), *Mercury* (Macrae *et al.*, 2008 ▸), *PLATON* (Spek, 2009 ▸) and *publCIF* (Westrip, 2010 ▸).

## supplementary crystallographic information

### Crystal data

**Table:**

C_14_H_16_NO^+^·C_4_H_6_NO_4_^−^·H_2_O	*F*(000) = 388
*M**_r_* = 364.39	*D*_x_ = 1.36 Mg m^−^^3^
Monoclinic, *P*2_1_	Cu *K*α radiation, λ = 1.54178 Å
Hall symbol: P 2yb	Cell parameters from 25 reflections
*a* = 18.310 (8) Å	θ = 28.2–34.6°
*b* = 5.2661 (10) Å	µ = 0.86 mm^−^^1^
*c* = 9.2792 (10) Å	*T* = 297 K
β = 96.070 (4)°	Rod, colorless
*V* = 889.7 (4) Å^3^	0.4 × 0.2 × 0.2 mm
*Z* = 2	

### Data collection

**Table:**

Enraf–Nonius CAD-4 diffractometer	1907 reflections with *I* > 2σ(*I*)
Radiation source: Enraf Nonius FR590	*R*_int_ = 0.020
Graphite monochromator	θ_max_ = 74.9°, θ_min_ = 2.4°
non–profiled ω/2τ scans	*h* = 0→22
Absorption correction: ψ scan (North *et al.*, 1968)	*k* = 0→6
*T*_min_ = 0.74, *T*_max_ = 0.86	*l* = −11→11
2114 measured reflections	3 standard reflections every 60 min
2051 independent reflections	intensity decay: 5%

### Refinement

**Table:**

Refinement on *F*^2^	Hydrogen site location: mixed
Least-squares matrix: full	H atoms treated by a mixture of independent and constrained refinement
*R*[*F*^2^ > 2σ(*F*^2^)] = 0.028	*w* = 1/[σ^2^(*F*_o_^2^) + (0.0517*P*)^2^ + 0.0743*P*] where *P* = (*F*_o_^2^ + 2*F*_c_^2^)/3
*wR*(*F*^2^) = 0.079	(Δ/σ)_max_ < 0.001
*S* = 1.06	Δρ_max_ = 0.17 e Å^−^^3^
2051 reflections	Δρ_min_ = −0.13 e Å^−^^3^
272 parameters	Extinction correction: (SHELXL2017; Sheldrick, 2015), Fc^*^=kFc[1+0.001xFc^2^λ^3^/sin(2θ)]^-1/4^
1 restraint	Extinction coefficient: 0.0104 (12)
0 constraints	Absolute structure: No quotients, so Flack (1983) parameter determined by classical intensity fit
Primary atom site location: structure-invariant direct methods	Absolute structure parameter: 0.1 (2)
Secondary atom site location: difference Fourier map	

### Special details

**Table:**

Geometry. All esds (except the esd in the dihedral angle between two l.s. planes) are estimated using the full covariance matrix. The cell esds are taken into account individually in the estimation of esds in distances, angles and torsion angles; correlations between esds in cell parameters are only used when they are defined by crystal symmetry. An approximate (isotropic) treatment of cell esds is used for estimating esds involving l.s. planes.

### Fractional atomic coordinates and isotropic or equivalent isotropic displacement parameters (Å^2^)

**Table:**

	*x*	*y*	*z*	*U*_iso_*/*U*_eq_	
C1B	0.86265 (9)	0.1336 (4)	0.25943 (17)	0.0285 (3)	
C2B	0.87228 (9)	0.3556 (4)	0.15574 (17)	0.0281 (3)	
H2B	0.834662	0.481729	0.171984	0.034*	
C3B	0.86125 (10)	0.2862 (4)	−0.00305 (18)	0.0331 (4)	
H3B1	0.872821	0.433561	−0.059083	0.040*	
H3B2	0.809703	0.247484	−0.028525	0.040*	
C4B	0.90650 (9)	0.0630 (4)	−0.04800 (18)	0.0309 (4)	
N1B	0.94481 (9)	0.4807 (4)	0.19193 (17)	0.0344 (3)	
O1B	0.90523 (7)	0.1301 (3)	0.37604 (13)	0.0363 (3)	
O2B	0.81052 (7)	−0.0126 (3)	0.22709 (14)	0.0410 (3)	
O3B	0.94788 (7)	−0.0504 (3)	0.04607 (14)	0.0378 (3)	
O4B	0.89947 (7)	0.0126 (3)	−0.18182 (14)	0.0437 (4)	
H1B1	0.9480 (15)	0.620 (7)	0.134 (3)	0.064 (8)*	
H1B2	0.9537 (12)	0.517 (5)	0.294 (3)	0.044 (6)*	
H1B3	0.9841 (16)	0.405 (7)	0.168 (3)	0.069 (9)*	
C1A	0.73892 (10)	0.5069 (4)	0.44043 (19)	0.0329 (4)	
H1A	0.768063	0.436672	0.367158	0.039*	
C2A	0.75763 (9)	0.3549 (4)	0.58118 (18)	0.0326 (4)	
H2A	0.741273	0.179814	0.561992	0.039*	
C3A	0.65856 (10)	0.4782 (4)	0.38426 (18)	0.0343 (4)	
C4A	0.63715 (12)	0.2766 (5)	0.2934 (2)	0.0458 (5)	
H4A	0.672227	0.164185	0.265603	0.055*	
C5A	0.56362 (13)	0.2416 (5)	0.2438 (3)	0.0547 (6)	
H5A	0.549539	0.104493	0.184080	0.066*	
C6A	0.51174 (12)	0.4086 (5)	0.2827 (3)	0.0562 (6)	
H6A	0.462589	0.385662	0.248470	0.067*	
C7A	0.53231 (12)	0.6099 (6)	0.3721 (3)	0.0578 (6)	
H7A	0.497042	0.722986	0.398421	0.069*	
C8A	0.60593 (11)	0.6449 (5)	0.4233 (2)	0.0450 (5)	
H8A	0.619641	0.781071	0.484053	0.054*	
C9A	0.72103 (9)	0.4489 (4)	0.70990 (17)	0.0300 (4)	
C10A	0.66288 (10)	0.3106 (4)	0.7532 (2)	0.0403 (4)	
H10A	0.646344	0.166838	0.701394	0.048*	
C11A	0.62902 (13)	0.3850 (6)	0.8737 (2)	0.0551 (6)	
H11A	0.589386	0.292688	0.900760	0.066*	
C12A	0.65359 (13)	0.5929 (6)	0.9524 (2)	0.0541 (6)	
H12A	0.631510	0.639855	1.034136	0.065*	
C13A	0.71124 (14)	0.7326 (5)	0.9101 (2)	0.0503 (5)	
H13A	0.728153	0.873940	0.963729	0.060*	
C14A	0.74423 (11)	0.6637 (4)	0.7880 (2)	0.0397 (4)	
H14A	0.782096	0.762143	0.758481	0.048*	
N1A	0.83958 (9)	0.3495 (5)	0.6135 (2)	0.0445 (4)	
O1A	0.76176 (8)	0.7615 (3)	0.46671 (15)	0.0392 (3)	
H1A1	0.8579 (18)	0.240 (8)	0.709 (3)	0.087 (11)*	
H1A2	0.8600 (15)	0.288 (7)	0.530 (3)	0.067 (9)*	
H1A3	0.8561 (15)	0.501 (7)	0.638 (3)	0.053 (8)*	
H1O1	0.7784 (15)	0.818 (7)	0.387 (3)	0.062 (8)*	
O1C	0.94824 (9)	0.6688 (3)	0.49070 (19)	0.0476 (4)	
H1OB	0.9297 (17)	0.820 (9)	0.453 (3)	0.077 (10)*	
H1OA	0.991 (2)	0.707 (9)	0.543 (4)	0.090 (11)*	

### Atomic displacement parameters (Å^2^)

**Table:**

	*U*^11^	*U*^22^	*U*^33^	*U*^12^	*U*^13^	*U*^23^
C1B	0.0317 (7)	0.0269 (8)	0.0277 (7)	−0.0005 (7)	0.0073 (6)	0.0007 (7)
C2B	0.0292 (7)	0.0260 (8)	0.0297 (7)	0.0032 (7)	0.0054 (6)	0.0030 (7)
C3B	0.0374 (8)	0.0338 (10)	0.0280 (7)	0.0067 (8)	0.0025 (6)	0.0061 (7)
C4B	0.0312 (7)	0.0305 (9)	0.0318 (7)	−0.0023 (7)	0.0075 (6)	0.0018 (7)
N1B	0.0367 (8)	0.0300 (8)	0.0367 (8)	−0.0035 (7)	0.0050 (6)	0.0064 (7)
O1B	0.0440 (7)	0.0328 (7)	0.0312 (6)	−0.0031 (6)	−0.0005 (5)	0.0062 (6)
O2B	0.0437 (7)	0.0411 (8)	0.0387 (6)	−0.0143 (7)	0.0061 (5)	0.0035 (6)
O3B	0.0425 (7)	0.0308 (7)	0.0406 (7)	0.0073 (6)	0.0068 (5)	0.0072 (6)
O4B	0.0458 (7)	0.0528 (10)	0.0328 (6)	0.0030 (7)	0.0054 (5)	−0.0066 (6)
C1A	0.0359 (9)	0.0326 (10)	0.0310 (8)	0.0018 (8)	0.0077 (6)	0.0030 (8)
C2A	0.0317 (8)	0.0334 (9)	0.0330 (8)	0.0023 (8)	0.0041 (6)	0.0039 (8)
C3A	0.0389 (9)	0.0338 (9)	0.0300 (7)	0.0022 (8)	0.0028 (6)	0.0045 (8)
C4A	0.0485 (11)	0.0399 (12)	0.0479 (11)	0.0043 (10)	0.0003 (8)	−0.0053 (10)
C5A	0.0568 (12)	0.0483 (13)	0.0561 (12)	−0.0057 (12)	−0.0075 (10)	−0.0066 (11)
C6A	0.0409 (11)	0.0594 (17)	0.0655 (14)	−0.0038 (11)	−0.0074 (10)	0.0067 (12)
C7A	0.0395 (10)	0.0593 (15)	0.0733 (15)	0.0108 (12)	−0.0004 (10)	−0.0058 (13)
C8A	0.0412 (10)	0.0426 (11)	0.0506 (11)	0.0057 (10)	0.0015 (8)	−0.0067 (10)
C9A	0.0299 (8)	0.0297 (9)	0.0298 (8)	0.0018 (7)	0.0013 (6)	0.0039 (7)
C10A	0.0378 (9)	0.0414 (11)	0.0427 (10)	−0.0083 (9)	0.0083 (7)	−0.0013 (9)
C11A	0.0513 (12)	0.0670 (17)	0.0506 (12)	−0.0069 (12)	0.0213 (9)	0.0014 (12)
C12A	0.0658 (13)	0.0590 (16)	0.0399 (10)	0.0156 (13)	0.0171 (9)	−0.0001 (11)
C13A	0.0746 (14)	0.0369 (11)	0.0380 (10)	0.0047 (11)	−0.0003 (9)	−0.0051 (9)
C14A	0.0471 (10)	0.0332 (10)	0.0382 (9)	−0.0060 (9)	0.0016 (7)	0.0010 (9)
N1A	0.0338 (8)	0.0613 (13)	0.0392 (9)	0.0097 (9)	0.0076 (6)	0.0119 (9)
O1A	0.0455 (7)	0.0349 (7)	0.0378 (7)	−0.0054 (6)	0.0065 (5)	0.0053 (6)
O1C	0.0472 (8)	0.0297 (8)	0.0625 (9)	−0.0008 (6)	−0.0098 (7)	−0.0011 (7)

### Geometric parameters (Å, º)

**Table:**

C1B—O2B	1.239 (2)	C5A—C6A	1.370 (4)
C1B—O1B	1.265 (2)	C5A—H5A	0.9300
C1B—C2B	1.536 (2)	C6A—C7A	1.374 (4)
C2B—N1B	1.488 (2)	C6A—H6A	0.9300
C2B—C3B	1.511 (2)	C7A—C8A	1.393 (3)
C2B—H2B	0.9800	C7A—H7A	0.9300
C3B—C4B	1.522 (3)	C8A—H8A	0.9300
C3B—H3B1	0.9700	C9A—C10A	1.384 (3)
C3B—H3B2	0.9700	C9A—C14A	1.386 (3)
C4B—O3B	1.246 (2)	C10A—C11A	1.391 (3)
C4B—O4B	1.263 (2)	C10A—H10A	0.9300
N1B—H1B1	0.92 (4)	C11A—C12A	1.365 (4)
N1B—H1B2	0.96 (2)	C11A—H11A	0.9300
N1B—H1B3	0.87 (3)	C12A—C13A	1.377 (4)
C1A—O1A	1.418 (2)	C12A—H12A	0.9300
C1A—C3A	1.516 (3)	C13A—C14A	1.387 (3)
C1A—C2A	1.539 (2)	C13A—H13A	0.9300
C1A—H1A	0.9800	C14A—H14A	0.9300
C2A—N1A	1.499 (2)	N1A—H1A1	1.08 (4)
C2A—C9A	1.513 (2)	N1A—H1A2	0.95 (3)
C2A—H2A	0.9800	N1A—H1A3	0.87 (4)
C3A—C8A	1.380 (3)	O1A—H1O1	0.88 (3)
C3A—C4A	1.386 (3)	O1C—H1OB	0.92 (4)
C4A—C5A	1.388 (3)	O1C—H1OA	0.90 (4)
C4A—H4A	0.9300		
			
O2B—C1B—O1B	125.99 (17)	C3A—C4A—H4A	119.8
O2B—C1B—C2B	117.27 (14)	C5A—C4A—H4A	119.8
O1B—C1B—C2B	116.47 (15)	C6A—C5A—C4A	120.2 (2)
N1B—C2B—C3B	110.56 (14)	C6A—C5A—H5A	119.9
N1B—C2B—C1B	110.74 (13)	C4A—C5A—H5A	119.9
C3B—C2B—C1B	114.48 (15)	C5A—C6A—C7A	120.0 (2)
N1B—C2B—H2B	106.9	C5A—C6A—H6A	120.0
C3B—C2B—H2B	106.9	C7A—C6A—H6A	120.0
C1B—C2B—H2B	106.9	C6A—C7A—C8A	120.1 (2)
C2B—C3B—C4B	115.70 (14)	C6A—C7A—H7A	119.9
C2B—C3B—H3B1	108.4	C8A—C7A—H7A	119.9
C4B—C3B—H3B1	108.4	C3A—C8A—C7A	120.2 (2)
C2B—C3B—H3B2	108.4	C3A—C8A—H8A	119.9
C4B—C3B—H3B2	108.4	C7A—C8A—H8A	119.9
H3B1—C3B—H3B2	107.4	C10A—C9A—C14A	118.70 (17)
O3B—C4B—O4B	125.39 (18)	C10A—C9A—C2A	118.39 (18)
O3B—C4B—C3B	119.12 (15)	C14A—C9A—C2A	122.89 (16)
O4B—C4B—C3B	115.47 (16)	C9A—C10A—C11A	120.5 (2)
C2B—N1B—H1B1	109.3 (17)	C9A—C10A—H10A	119.8
C2B—N1B—H1B2	111.4 (14)	C11A—C10A—H10A	119.8
H1B1—N1B—H1B2	114 (3)	C12A—C11A—C10A	120.4 (2)
C2B—N1B—H1B3	119 (2)	C12A—C11A—H11A	119.8
H1B1—N1B—H1B3	96 (3)	C10A—C11A—H11A	119.8
H1B2—N1B—H1B3	107 (2)	C11A—C12A—C13A	119.7 (2)
O1A—C1A—C3A	114.28 (16)	C11A—C12A—H12A	120.2
O1A—C1A—C2A	108.10 (15)	C13A—C12A—H12A	120.2
C3A—C1A—C2A	111.15 (14)	C12A—C13A—C14A	120.4 (2)
O1A—C1A—H1A	107.7	C12A—C13A—H13A	119.8
C3A—C1A—H1A	107.7	C14A—C13A—H13A	119.8
C2A—C1A—H1A	107.7	C9A—C14A—C13A	120.3 (2)
N1A—C2A—C9A	111.47 (15)	C9A—C14A—H14A	119.8
N1A—C2A—C1A	107.92 (15)	C13A—C14A—H14A	119.8
C9A—C2A—C1A	114.98 (16)	C2A—N1A—H1A1	113.1 (18)
N1A—C2A—H2A	107.4	C2A—N1A—H1A2	108.5 (16)
C9A—C2A—H2A	107.4	H1A1—N1A—H1A2	111 (3)
C1A—C2A—H2A	107.4	C2A—N1A—H1A3	110.3 (18)
C8A—C3A—C4A	119.11 (18)	H1A1—N1A—H1A3	102 (3)
C8A—C3A—C1A	121.73 (18)	H1A2—N1A—H1A3	112 (3)
C4A—C3A—C1A	119.16 (18)	C1A—O1A—H1O1	107 (2)
C3A—C4A—C5A	120.4 (2)	H1OB—O1C—H1OA	106 (4)
			
O1A—C1A—C2A—N1A	−65.0 (2)	C5A—C6A—C7A—C8A	0.1 (4)
O1A—C1A—C2A—C9A	60.14 (19)	C6A—C7A—C8A—C3A	0.2 (4)
C3A—C1A—C2A—N1A	168.86 (17)	C2A—C9A—C10A—C11A	177.95 (19)
C3A—C1A—C2A—C9A	−66.1 (2)	C14A—C9A—C10A—C11A	−0.6 (3)
O1A—C1A—C3A—C4A	149.76 (18)	C2A—C9A—C14A—C13A	−176.29 (19)
O1A—C1A—C3A—C8A	−31.4 (2)	C10A—C9A—C14A—C13A	2.2 (3)
C2A—C1A—C3A—C4A	−87.6 (2)	C9A—C10A—C11A—C12A	−1.2 (3)
C2A—C1A—C3A—C8A	91.3 (2)	C10A—C11A—C12A—C13A	1.4 (4)
N1A—C2A—C9A—C10A	−131.49 (19)	C11A—C12A—C13A—C14A	0.2 (4)
N1A—C2A—C9A—C14A	47.0 (3)	C12A—C13A—C14A—C9A	−2.0 (3)
C1A—C2A—C9A—C10A	105.3 (2)	O1B—C1B—C2B—N1B	17.4 (2)
C1A—C2A—C9A—C14A	−76.2 (2)	O1B—C1B—C2B—C3B	143.24 (16)
C1A—C3A—C4A—C5A	178.1 (2)	O2B—C1B—C2B—N1B	−168.28 (16)
C8A—C3A—C4A—C5A	−0.7 (3)	O2B—C1B—C2B—C3B	−42.5 (2)
C1A—C3A—C8A—C7A	−178.7 (2)	N1B—C2B—C3B—C4B	73.0 (2)
C4A—C3A—C8A—C7A	0.2 (3)	C1B—C2B—C3B—C4B	−53.0 (2)
C3A—C4A—C5A—C6A	1.0 (4)	C2B—C3B—C4B—O3B	1.4 (3)
C4A—C5A—C6A—C7A	−0.6 (4)	C2B—C3B—C4B—O4B	−177.08 (16)

### Hydrogen-bond geometry (Å, º)

*CgB* is the centroid of phenyl ring *B* (C9–C14).

**Table:**

*D*—H···*A*	*D*—H	H···*A*	*D*···*A*	*D*—H···*A*
N1*B*—H1*B*1···O3*B*^i^	0.92 (4)	1.92 (4)	2.819 (2)	168 (3)
N1*B*—H1*B*3···O3*B*^ii^	0.87 (3)	2.46 (3)	3.112 (2)	132 (3)
N1*B*—H1*B*3···O4*B*^ii^	0.87 (3)	2.20 (3)	2.868 (2)	134 (3)
O1*A*—H1*O*1···O2*B*^i^	0.88 (3)	1.88 (3)	2.752 (2)	171 (3)
N1*A*—H1*A*1···O4*B*^iii^	1.08 (4)	1.70 (4)	2.742 (3)	162 (3)
N1*A*—H1*A*2···O1*B*	0.95 (3)	1.92 (3)	2.862 (2)	173 (3)
O1*C*—H1*OB*···O1*B*^i^	0.92 (4)	1.82 (4)	2.734 (2)	173 (3)
O1*C*—H1*OA*···O1*B*^iv^	0.90 (4)	2.01 (4)	2.840 (2)	153 (4)
N1*A*—H1*A*3···O1*C*	0.87 (4)	2.45 (3)	2.926 (3)	115 (2)
N1*B*—H1*B*2···O1*C*	0.96 (2)	2.01 (3)	2.938 (2)	163 (2)
C2*A*—H2*A*···O1*A*^v^	0.98	2.42	3.304 (3)	150
C14*A*—H14*A*···O4*B*^vi^	0.93	2.53	3.371 (3)	150
C3*B*—H3*B*2···CgB^iii^	0.97	2.87	3.6127 (16)	134

Symmetry codes: (i) *x*, *y*+1, *z*; (ii) −*x*+2, *y*+1/2, −*z*; (iii) *x*, *y*, *z*+1; (iv) −*x*+2, *y*+1/2, −*z*+1; (v) *x*, *y*−1, *z*; (vi) *x*, *y*+1, *z*+1.
